# Does bone mobilization interfere with energy metabolism in transition cows?

**DOI:** 10.3168/jdsc.2022-0239

**Published:** 2022-09-03

**Authors:** M.O. Matthaei, S.U. Kononov, J. Rehage, G. Szura, I. Leiter, K. Hansen, S. Daenicke, D. von Soosten, S. Kersten, Ulrich Meyer, M.R. Wilkens

**Affiliations:** 1 Institute of Animal Nutrition, Nutrition Diseases and Dietetics, Faculty of Veterinary Medicine, University of Leipzig, 01403 Leipzig, Saxony, Germany; 2 Clinic for Cattle, University of Veterinary Medicine Hannover, Foundation, 30173 Hannover, Lower Saxony, Germany; 3 Institute of Physiology and Cell Biology, University of Veterinary Medicine Hannover, Foundation, 30173 Hannover, Lower Saxony, Germany; 4 Institute of Animal Nutrition, Federal Research Institute for Animal Health, 38116 Braunschweig, Lower Saxony, Germany

## Abstract

The onset of lactation represents a challenge for both mineral homeostasis and energy metabolism in high-performing dairy cows. It has been shown that subclinical and clinical hypocalcemia increases the risk of ketosis and recent studies suggest that bone-derived endocrine factors could play a role in intermediary metabolism. Therefore, we analyzed serum samples from calculated d −7, calculated d −3, d +1, d +3, and d +7 relative to calving from 15 multiparous cows for total Ca, the bone resorption marker CrossLaps, the bone formation marker intact osteocalcin, undercarboxylated osteocalcin (ucOC), insulin, glucose, nonesterified fatty acids, β-hydroxybutyrate, and insulin-like growth factor 1. Serum concentrations of Ca on d −3 and d +1 were associated with parameters of energy metabolism on d +3 and d +7. As we found large variations for serum concentrations of ucOC already on d −7, we allocated the cows retrospectively to 3 groups: low ucOC, medium ucOC, and high ucOC. These groups differed not only in their ucOC dynamics, but also in insulin sensitivity estimated using the revised quantitative insulin sensitivity index (RQUICKI). High ucOC cows presented with the highest RQUICKI throughout the entire observation period. Our data further support the hypothesis that low serum Ca precedes disturbances of energy metabolism. Furthermore, from our preliminary results it can be assumed that the potential link between mineral homeostasis, bone turnover, and intermediary metabolism should be further investigated.

## Highlights

• Low serum Ca in dairy cows seems to precede imbalances of intermediary metabolism.
• Calcium homeostasis and energy metabolism might be linked by bone-derived factors.
• Prepartum serum concentration of osteocalcin varies substantially in dairy cows.
• Calcium and osteocalcin around parturition could be predictive for metabolic stability.

## Graphical Abstract

**Figure:**
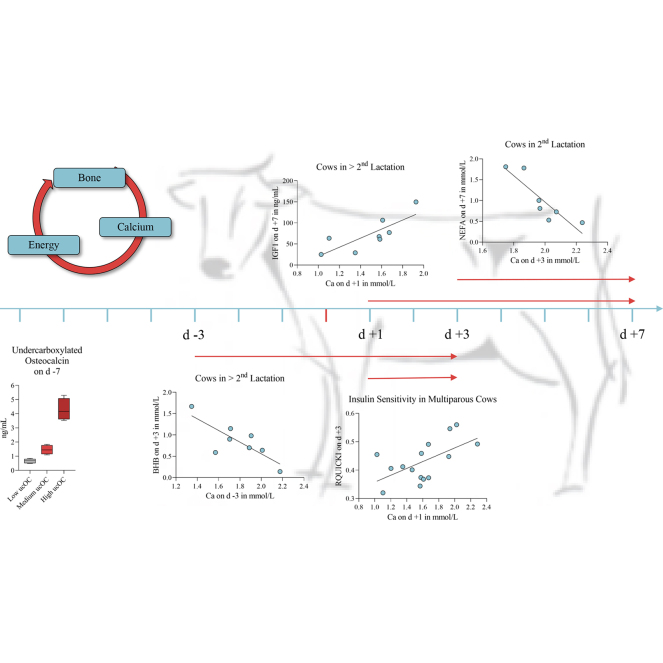
**Summary:** We investigated the interaction between mineral homeostasis and energy metabolism indicated by correlations between plasma Ca and parameters related to intermediary metabolism such as insulin-like growth factor 1 (IGF1), nonesterified fatty acids (NEFA), β-hydroxybutyrate (BHB), and the revised quantitative insulin sensitivity index (RQUICKI). Our results indicate that disturbances of peripartum Ca homeostasis precede metabolic imbalances. In addition to the already known links, the bone-derived endocrine factor undercarboxylated osteocalcin (ucOC) might play a significant role in this context. In this preliminary study, we could distinguish three different types of cows according to their prepartum plasma concentration of ucOC. Intriguingly, these groups differed with respect to insulin sensitivity throughout the entire observation period.

High-yielding transition cows experience a negative energy balance (**NEB**) in early lactation. Due to impaired hepatic synthesis, serum concentrations of IGF1 decline dramatically. This represents the so-called uncoupling of the somatotropic axis that enables growth hormone (**GH**) to act independent of IGF1 and to stimulate lipolysis and gluconeogenesis and is correlated with the severity of the NEB (Ingvartsen et al., 2003; Fenwick et al., 2008). In addition, insulin resistance mediates nutrient partitioning toward the mammary gland (Horst et al., 2005; Baumgard et al., 2017).

At the same time, calcium (Ca) homeostasis is severely challenged. Dairy cows experiencing subclinical hypocalcemia (**SHC**) defined as serum Ca <2.0 m*M* within 48 h postpartum presented with significantly higher serum concentrations of nonesterified fatty acids (**NEFA**) in comparison to normocalcemic cows (Reinhardt et al., 2011). This observation, as well as the fact that SHC increases the animals' risk to develop ketosis (Rodríguez et al., 2017), is often explained by the reduction in feed intake caused by SHC (Martinez et al., 2014). However, there are several indications that the interaction between Ca homeostasis and energy metabolism might be far more complex. For example, SHC was shown to exert an effect on insulin secretion (Martinez et al., 2014). On the other hand, low serum concentrations of IGF1 in growing goats fed a diet restricted in CP were associated with a downregulation of renal RNA expression of 1α-hydroxylase, the enzyme crucial for the hydroxylation of 25-hydroxycholecalciferol, resulting in decreased serum concentrations of 1,25-dihydroxycholecalciferol, even when Ca homeostasis was additionally challenged by restricted Ca supply (Wilkens et al., 2018). For the fresh cow, this observation might indicate that the pronounced imbalance between intake and demand of protein and energy in early lactation that is accompanied by low serum IGF1 could aggravate Ca homeostasis not only by a reduction in DMI but also via an inhibition of the endocrine response that should restore Ca homeostasis.

When normocalcemic cows were compared with animals that developed clinical or subclinical hypocalcemia, it seemed that the timely onset of bone mobilization plays a key role in the first response to the increased Ca demand. Calcitonin, the hormone that inhibits bone mobilization, was increased in cows developing SCH, and in animals with milk fever, the rise in the bone resorption marker hydroxyproline was not as pronounced as in normocalcemic cows at parturition (Rodríguez et al., 2016; Hyde et al., 2019). Cows that develop subclinical or clinical hypocalcemia in association with presumably insufficient bone mobilization present with lower rumination rate, lower DMI, and consequently lower intake of both energy and Ca (Goff et al., 2020), thus entering a vicious circle of hypocalcemia and NEB.

However, there are some hints that bone mobilization and intermediary metabolism might not only be linked indirectly but also directly. Interestingly, an infusion of parathyroid hormone that induces bone mobilization had no effect on plasma GH but increased the hepatic production of IGF1 in calves. As an infusion of Ca alone did not affect IGF1 and calcitonin decreased plasma Ca, GH, and IGF1, it might be speculated that bone-derived factors are involved in hepatic IGF1 synthesis (Coxam et al., 1990). Furthermore, calving is accompanied by a decrease in the bone formation marker osteocalcin (**OC**; Wilkens et al., 2012; Rodney et al., 2018b). The latter observation has gained a lot of attention as studies in rodents suggest that OC, especially in its undercarboxylated form (**ucOC**), has endocrine functions. A gain-of-function mutation in mice resulted in decreased plasma glucose and increased insulin sensitivity (Lee et al., 2007) and administration of ucOC to mice kept on a high-fat diet had a beneficial effect on glucose tolerance and prevented hepatic steatosis (Ferron et al., 2012). Comparable results were obtained in aged laying hens in which a treatment with ucOC reduced the impact of a high-fat diet on insulin sensitivity and hepatic expression of inflammatory cytokines and reduced oxidative stress (Wu et al., 2021). In contrast, very recent studies in genetically modified mice and rats did not show any effects of OC or ucOC on carbohydrate metabolism (Diegel et al., 2020; Moriishi et al., 2020).

Nevertheless, the idea that energy and bone metabolism are directly linked and that these interactions could play an important role in the periparturient cow that has to mobilize bone tissue while being in a NEB is intriguing. Therefore, this preliminary study aims to explore potential interactions between Ca homeostasis, OC, and intermediary metabolism in transition cows.

Part of the data and samples for this study were retrieved from a previously published trial on the effects of vitamin E (**Vit E**) and CLA on performance, lipomobilization, and energy metabolism in dairy cows that was carried out at the experimental station of the Federal Research Institute for Animal Health, Brunswick, Germany, in accordance with the German Animal Welfare Act and was approved by the Lower Saxony State Office for Consumer Protection and Food Safety (LAVES, Oldenburg, Germany). Schäfers et al. (2017) described the experimental design, diets, collection of samples, analytical procedures, and recordings of performance data and their further processing in detail. Briefly, 64 pluriparous German Holstein cows were allocated into 4 groups (n = 16/group): 3 treatment groups (CLA, Vit E, and CLA+Vit E) and 1 control group. Cows were fed ad libitum with a standardized partial mixed ration from self-feeding troughs (RIC, Insentec B.V.). Additionally, the animals were supplied with 3 kg/d per cow of concentrate by means of automated self-feeding stations (Insentec B.V.). The ration consisted of 60% concentrate and 40% silage (50% corn, 50% grass silage on a DM basis) from d −42 until parturition. After parturition, the portion of concentrate steadily increased from 30% to 50% until d +21.

Since no effects of any treatment were found for parameters of Ca homeostasis, bone mobilization, and energy metabolism in the subgroup of cows used for the present study, the data set appeared suitable for further evaluation. In a first approach to investigate physiological profiles of parameters of bone and energy metabolism, we analyzed serum samples from calculated d −7 (d −10 to d −5), calculated d −3 (d −5 to d −2), d +1, d +3, and d +7 relative to calving from 15 multiparous cows (7 cows entering their second lactation, 4 cows entering their third lactation, 4 cows entering their fourth or higher lactations) for total Ca, the bone resorption marker CrossLaps (**CL**), intact OC (**iOC**), ucOC, insulin, glucose, NEFA, BHB, and IGF1.

The serum concentrations of Ca were measured colorimetrically by a standard spectrometric analysis (Sarkar and Chauhan, 1967). Analysis of CL, iOC, and ucOC was done using the following commercially available ELISA kits: Serum CrossLaps ELISA (Immundiagnostic System GmbH), MicroVue Osteocalcin EIA Kit (Quidel Corp.), and Undercarboxylated Osteocalcin (Glu-OC) MK118 (Clontech Labs, Takara Bio Inc.) according to the manufacturers' instructions. Intra- and interassay coefficients of variance in our laboratory were 15.2% and 7.7% for CL, 6.5% and 6.0% for iOC, and 5.3% and 2.2% for ucOC, respectively.

Serum insulin and IGF1 were measured using RIA kits (IM3210 Insulin IRMA and A15729 IGF-I IRMA, Beckman Coulter). Analyses for glucose, NEFA, and BHB were performed by a photometric method using the Eurolyser CCA 180 (Eurolyser Diagnostica GmbH). Insulin sensitivity was estimated by calculating the revised quantitative insulin sensitivity index (**RQUICKI**) according to Holtenius and Holtenius (2007) using the following formula: RQUICKI = 1/[log glucose in mg/dL + log insulin in μU/mL + log NEFA in mmol/L].

Kolmogorov-Smirnov test was used to test for normal distribution. Data that were not normally distributed (CL, iOC, ucOC, insulin, NEFA, BHB, IGF1) were log-transformed before further analysis. We conducted a repeated measures ANOVA corrected according to Geisser-Greenhouse if sphericity could not be assumed. The model used to reveal potential effects of time and lactation number on the different plasma parameters related to mineral homeostasis, bone turnover, and intermediary metabolism included lactation number, time (experimental day relative to parturition), and the interaction between lactation number and time as fixed effects and the cow as subject. For ucOC and RQUICKI, an additional statistical model was calculated with the ucOC groups (low ucOC, medium ucOC, and high ucOC) and time as fixed effects. In case of significant differences of time, lactation number, or ucOC group, Bonferroni's post-test adjusted for multiple comparisons was used to verify differences over time or due to either lactation number or ucOC group. According to data distribution, data are presented either as mean ± standard deviation or as median and the 25% percentile and the 75% percentile given in parentheses.

Depending on the distribution of data, we calculated correlation coefficients (r) according to either Pearson or Spearman to reveal associations of parameters on the same day or on different days. In some cases, this was done separately for cows entering the second lactation and older animals. All analyses were done using GraphPad Prism 9.1.2 (GraphPad Software); no samples were excluded from the data.

Serum concentrations of Ca, CL, iOC, ucOC, insulin, glucose, NEFA, BHB, and IGF1 around parturition are given in Table 1. As expected, we observed a significant, transient drop in serum Ca at the onset of lactation that was accompanied by a rise in the bone resorption marker CL and a decrease in iOC and ucOC indicating bone mobilization as a response to the demand of Ca for milk production. In addition, there was an increase in both NEFA and BHB after calving, whereas serum concentrations of IGF1 and insulin decreased significantly. These results represent the above-mentioned adaptation to lactation and similar dynamics have been published before (Reist et al., 2003; Rodney et al., 2018a).

**Table 1:** Serum concentrations of parameters related to mineral homeostasis and energy metabolism around parturition (days related to calving, prepartum as calculated) presented as means ± SD in case of normal distribution or as median and the 25% and 75% percentiles given in parentheses (data from 15 multiparous cows)^1^

Item	Day −7	Day −3	Day +1	Day +3	Day +7	T	LN	T × LN
Calcium (mmol/L)	2.18 ± 0.34^a^	1.88 ± 0.33a,b	1.60 ± 0.35^b^	2.02 ± 0.25^a^	2.15 ± 0.29^a^	<0.0001 (32.3%)	0.0720	0.5681
CrossLaps (ng/mL)	0.25^a^	0.21^a^	0.50^b^	0.67^b^	0.65^b^	<0.0001 (39.5%)	0.2362	0.6365
	(0.18; 0.41)	(0.16; 0.41)	(0.41; 0.67)	(0.46; 0.76)	(0.53; 0.93)			
Intact osteocalcin (ng/mL)								
All cows (n = 15)	36.8	40.8*	22.9*	25.6	33.2	0.0009 (13.7%)	0.0045 (23.3%)	0.1357
	(33.3; 45.8)	(26.2; 52.1)	(16.2; 39.9)	(18.9; 36.8)	(24.6; 43.8)			
Second lactation (n = 7)	38.8	44.9	39.9	35.1	43.2			
	(33.7; 63.5)	(43.7; 58.9)	(28.0; 44.8)	(25.6; 37.0)	(29.4; 44.2)			
>Second lactation (n = 8)	34.1 ± 8.49^a^	29.6 ± 13.5^ab^	17.5 ± 5.28^b^	23.3 ± 9.47^ab^	30.6 ± 10.8^a^			
Undercarboxylated osteocalcin (ng/mL)	1.18^a^	1.08^a^	0.74^b^	0.66^b^	0.84^ab^	0.0008 (3.7%)	0.1302	0.0281 (1.4%)
	(0.76; 3.53)	(0.84; 3.41)	(0.59; 3.12)	(0.44; 2.93)	(0.56; 2.77)			
Insulin (μIU/mL)	21.3^a^	17.5^ab^	10.0^bc^	7.17^c^	5.42^c^	<0.0001 (40.3%)	0.7636	0.0142 (9.0%)
	(15.3; 35.1)	(14.5; 22.9)	(4.60; 19.0)	(4.75; 13.8)	(4.00; 7.88)			
Glucose (mmol/L)	2.51 ± 0.47	2.72 ± 0.53	2.91 ± 0.91	2.70 ± 0.61	39.2 ± 2.18	0.0592	0.7948	0.1299
Nonesterified fatty acids (mmol/L)								
All cows (n = 15)	0.18	0.26*	0.60*	0.57	0.73	<0.0001 (41.5%)	0.0055 (7.2%)	0.0004 (14.2%)
	(0.15; 0.36)	(0.16; 0.46)	(0.32; 0.99)	(0.47; 0.98)	(0.53; 0.93)			
Second lactation (n = 7)	0.15^a^	0.16^a^	0.32^ab^	0.52^bc^	0.81^c^			
	(0.12; 0.29)	(0.13; 0.26)	(0.23; 0.52)	(0.47; 0.98)	(0.53; 1.78)			
>Second lactation (n = 8)	0.20^a^	0.45^ab^	0.98^c^	0.60^bc^	0.59^bc^			
	(0.16; 0.49)	(0.29; 0.77)	(0.70; 1.34)	(0.39; 1.15)	(0.52; 0.80)			
BHB (mmol/L)	0.55^a^	0.59^ab^	0.76^ab^	0.86^ab^	0.87^b^	0.0335 (16.9%)	0.4508	0.7613
	(0.48; 0.64)	(0.48; 0.66)	(0.69; 1.11)	(0.64; 1.01)	(1.23; 0.93)			
IGF1 (ng/mL)	193^a^	140^a^	76.8^b^	84.3^bc^	61.3^c^	<0.0001 (44.5%)	0.4465	0.0810
	(147; 204)	(109; 217)	(51.1; 126)	(36.5; 119)	(29.1; 77.1)			

a–c Different superscripts indicate significant (*P* < 0.05) differences between the sampling times.

1 *P*-values for the factors time (T), lactation number (LN), or their interaction (T × LN) and in case of significant differences the contribution of total variance in % revealed by repeated measures ANOVA.

* Asterisks indicate differences between second and >second lactation as revealed by Bonferroni's post-test adjusted for multiple comparisons.

Intact OC was significantly influenced by parity (second lactation vs. >second lactation, *P* = 0.0045). This is in contrast to the study done by Viera-Neto et al. who found no effect of parity on iOC and ucOC (Vieira-Neto et al., 2021). Rodney et al. (2018b) compared primiparous and multiparous cows; they found a significant difference with respect to iOC and a trend for greater ucOC concentrations in primiparous animals.

In cows in greater than second lactation (n = 8), serum Ca on d −3 correlated with serum concentrations of glucose (r: 0.859, *P* = 0.0063) and BHB (r: −0.794, *P* = 0.0188) on d +3. Furthermore, serum concentrations of Ca on d +1 correlated with IGF1 (r: 0.801, *P* = 0.0169) on d +7. Looking at all animals enrolled, we found a weaker but significant correlation between Ca on d +1 and RQUICKI on d +3 (r: 0.588, *P* = 0.0212). Lean et al. (2014) also reported a positive correlation of glucose and a negative correlation of BHB with serum Ca 7 d earlier. These results further support the hypothesis that peripartum Ca homeostasis is linked to intermediary metabolism. In addition, as the challenge of Ca homeostasis seems to precede alterations of parameters related to energy metabolism at least in cows in greater than second lactation, animals at risk might be identified already before or immediately after parturition.

In animals entering their second lactation (n = 7), we found correlations between Ca on d +3 with NEFA (r: −0.880, *P* = 0.0090) and RQUICKI (r: 0.814, *P* < 0.0257) on d +7. Again, from these results it might be speculated that serum Ca concentrations might be predictive for imbalances of energy metabolism later on. The association between SCH and an increased risk of ketosis has been shown in several studies (Reinhardt et al., 2011; Rodríguez et al., 2017). A link between serum Ca on d +1 and d +3 and ketosis has also been described recently by Venjakob et al. (2021), but longitudinal studies starting before parturition and investigating the exact time patterns are scarce.

Independent of age and iOC, we found large variations for serum ucOC already before parturition (Table 1). Therefore, we allocated the cows retrospectively to 3 groups with median serum concentrations of ucOC of 0.72 (0.52; 0.93) ng/mL, 1.70 ng/mL (1.13; 1.80), and 4.16 ng/mL (3.61; 5.09) on d −7 (low ucOC, medium ucOC, and high ucOC). High ucOC (n = 4) and low ucOC (n = 6) cows showed more or less stable plasma concentrations of ucOC throughout the entire observation period, whereas the concentrations in medium ucOC (n = 5) animals decreased after calving (Figure 1A). We revealed a significant effect of group for RQUICKI (*P* = 0.0136). In the low ucOC group, RQUICKI was lowest antepartum and increased postpartum, whereas medium ucOC cows showed an opposite pattern, and high ucOC animals were characterized by the highest RQUICKI antepartum and postpartum (Figure 1B). In a very recent study done in humans, low serum concentrations of OC in late pregnancy were associated with an impaired response to an oral glucose tolerance test postpartum in patients with gestational diabetes mellitus (Gong et al., 2022). Lean et al. (2014) suggested already in 2014 that bone and intermediary metabolism are linked in dairy cows, too. Since then, results on the role of OC in energy metabolism are inconsistent. In contrast to Lee et al. (2007), Diegel et al. (2020) and Moriishi et al. (2020) could not find any alterations of insulin secretion and insulin sensitivity OC knockout mice. In postmenopausal women, Ugurlu et al. (2022) reported lower serum concentrations of OC in patients with metabolic syndrome compared with a control group. Lower ucOC concentrations in comparison to healthy controls were also demonstrated in hyperglycemic humans and patients diagnosed with type 2 diabetes (Arponen et al., 2020).

**Figure 1:**
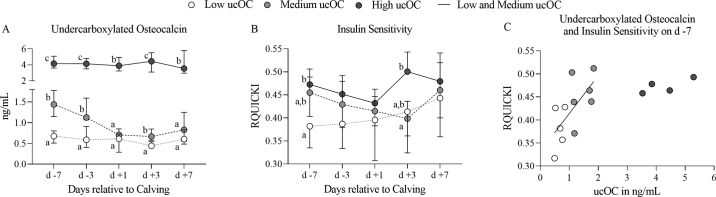
Based on their serum concentrations of undercarboxylated osteocalcin (ucOC) on d −7 relative to calving, animals were retrospectively allocated to 3 groups (low ucOC, medium ucOC, and high ucOC). (A) Time pattern of serum ucOC (median and the 25% and 75% percentiles), (B) revised quantitative insulin sensitivity index (RQUICKI) in the 3 groups (means ± SD), and (C) correlation between ucOC and RQUICKI in cows of the low ucOC and the medium ucOC group. Results of the statistical analysis are given in the text; different letters indicate significantly different medians or means on the respective day.

Although no differences in RQUICKI were found on d +7, our results might represent differences in the dynamics of adaptation to lactation. The high variances in ucOc (Rodney et al., 2018a,b) could corroborate the idea that different “ucOC groups” can be found within a population of dairy cows. At least, cows of the high ucOC group seem to show some peculiarities; the correlation between ucOC and RQUICKI revealed in animals belonging to the low ucOc and the medium ucOc groups (r: 0.727, *P* = 0.0144) was not demonstrated in the high ucOC group. Because the number of animals in our preliminary study was very low, further investigations are needed to verify whether the association that is suggested by the data really differs from that seen in the low ucOC and medium ucOC groups (Figure 1C). With respect to the correlations over time, it has to be pointed out that these are subject to the influence of autocorrelation. In addition, the parameters investigated are affected by several different factors and feedback mechanisms. Future studies should therefore be carefully planned to differentiate between cause, consequence, and coincidence.

From our preliminary data it can be concluded that low serum Ca precedes disturbances of energy metabolism. The role of ucOC and its potential endocrine functions seems to be more complex, but it might turn out to be an interesting factor involved in the development of metabolic disturbances in early lactation.
